# Lobish: Symbolic Language for Interpreting Electroencephalogram Signals in Language Detection Using Channel-Based Transformation and Pattern

**DOI:** 10.3390/diagnostics14171987

**Published:** 2024-09-08

**Authors:** Turker Tuncer, Sengul Dogan, Irem Tasci, Mehmet Baygin, Prabal Datta Barua, U. Rajendra Acharya

**Affiliations:** 1 Department of Digital Forensics Engineering, Technology Faculty, Firat University, 23119 Elazig, Türkiye; turkertuncer@firat.edu.tr; 2 Department of Neurology, School of Medicine, Firat University, 23119 Elazig, Türkiye; itasci@firat.edu.tr; 3 Department of Computer Engineering, Faculty of Engineering and Architecture, Erzurum Technical University, 25050 Erzurum, Türkiye; mehmet.baygin@erzurum.edu.tr; 4 School of Business (Information System), University of Southern Queensland, Springfield, QLD 4350, Australia; prabal.barua@usq.edu.au; 5 School of Mathematics, Physics and Computing, University of Southern Queensland, Springfield, QLD 4350, Australia; rajendra.acharya@usq.edu.au

**Keywords:** ChannelPat, channel-based signal transformation, Lobish, tkNN, EEG language detection, advanced signal processing, feature engineering

## Abstract

Electroencephalogram (EEG) signals contain information about the brain’s state as they reflect the brain’s functioning. However, the manual interpretation of EEG signals is tedious and time-consuming. Therefore, automatic EEG translation models need to be proposed using machine learning methods. In this study, we proposed an innovative method to achieve high classification performance with explainable results. We introduce channel-based transformation, a channel pattern (ChannelPat), the t algorithm, and Lobish (a symbolic language). By using channel-based transformation, EEG signals were encoded using the index of the channels. The proposed ChannelPat feature extractor encoded the transition between two channels and served as a histogram-based feature extractor. An iterative neighborhood component analysis (INCA) feature selector was employed to select the most informative features, and the selected features were fed into a new ensemble k-nearest neighbor (tkNN) classifier. To evaluate the classification capability of the proposed channel-based EEG language detection model, a new EEG language dataset comprising Arabic and Turkish was collected. Additionally, Lobish was introduced to obtain explainable outcomes from the proposed EEG language detection model. The proposed channel-based feature engineering model was applied to the collected EEG language dataset, achieving a classification accuracy of 98.59%. Lobish extracted meaningful information from the cortex of the brain for language detection.

## 1. Introduction

Language detection is an increasingly important field of research in today’s global and multilingual world [1]. Automatic language detection systems have critical applications in various domains [2]. For instance, they are used in multilingual customer services for accurate language routing, language-based analyses of social media content, online content filtering and classification, and even in national security applications for detecting suspicious communications. While traditional language detection methods typically work with text or audio data [3,4,5], detecting language using brain signals is a novel and promising approach.

Language detection plays a crucial role in various applications across different sectors. Multilingual customer services enable the automatic routing of customer calls to representatives who speak the appropriate language, enhancing customer experience and operational efficiency [6]. For social media platforms and content providers, language detection facilitates automatic categorization and analyses of content in different languages, allowing for better content management and targeted marketing [7]. In the field of machine translation, accurate language detection significantly improves the efficiency and accuracy of translation systems [8]. Libraries and archives benefit from language detection for the automatic classification and indexing of multilingual documents, making vast collections more accessible [9]. Educational technologies leverage language detection to identify the user’s native language and the language being learned, personalizing the learning experience in language learning applications [10]. Moreover, in the realm of cybersecurity, language detection aids in the analysis of suspicious communications, contributing to threat detection and prevention efforts [11]. These diverse applications underscore the importance of accurate and efficient language detection systems in our increasingly interconnected and multilingual world.

In recent years, the analysis of brain signals using brain–computer interfaces (BCIs) has become very popular [12]. An electroencephalogram (EEG) measures the electrical activities of brain waves, individuals’ cognitive, emotional, linguistic, and neurological states [13,14]. EEG signals are generally used for a diagnosis in the field of neurology to detect schizophrenia, depression, and Parkinson’s disease [15]. Of the recent advances in artificial intelligence, they have significantly contributed to the automatic classification of EEG signals [16,17]. Many artificial intelligence-based models have been developed to capture hidden patterns in EEG signals and successfully perform various classification tasks in BCI-based systems [18]. Language is a basic tool with its own rules and structures that people use to communicate and express themselves [19]. Every written and spoken language has its own structure, words, syllables, and accents [20]. Therefore, there are differences in brain signals when a native speaker encounters a sentence that they have seen or heard in their own language. Automatic language detection is a process that aims to identify the language a person is thinking or speaking through EEG data [21]. In this way, it aims to understand the neuroscientific basis of language learning processes and to develop new solutions in areas such as language therapy, language learning, and language education [22,23].

In this work, a new machine learning model has been developed for automatic language detection using EEG signals. To test the model, EEG signals were collected from Arabic and Turkish native speakers. From these collected signals, a feature vector is extracted using a new feature extractor called ChannelPat and the dimensionality of the feature vector is reduced using the INCA method. In the last stage of the model, a new ensemble kNN method is proposed and the collected signals are classified. The results show that EEG signals and the ChannelPat method can be used for automatic language detection with high accuracy and precision.

Recognizing the need for explainable models, we introduced Lobish, a symbolic language designed to provide interpretable results by encoding and interpreting EEG signals in a clear and structured manner. Lobish uses four letters, each representing a different brain lobe, enabling neuroscientific explanations of brain activity during language processing.

Given the multi-channel nature of EEG signals, we proposed two specific channel-based models to enhance classification performance. These models take advantage of the distinct patterns observed across different channels, leading to more accurate language detection.

To test the proposed models, we created a new EEG language dataset by collecting signals from native speakers of Arabic and Turkish. This dataset was essential in evaluating the effectiveness of our approach.

### 1.1. Literature Review

Language detection is not one of the most frequently studied topics in the literature [24]. Especially in this field, there is no dataset available. Existing EEG and language-based studies focus on letter/syllable/word/sentence recognition and native speaker detection. In this context, Table 1 summarizes the studies that use EEG and language-based machine learning models.

As shown in Table 1, studies in the literature on language using EEG signals are mostly focused on word/sentence recognition [26,28,32]. Only a limited number of native language recognition studies are available [24,25]. This work presents a new contribution to the literature and develops a native speaker detection model.

### 1.2. Literature Gaps

The research gaps based on the literature review are given below:

- Deep learning models are widely employed by the researchers. As a result, many deep learning-based models are used to classify the EEG signals with high classification performance. However, these deep learning models often have high time complexities [34].
- There is a limited number of explainable models in this area. Most models have focused solely on classification performance, neglecting the interpretability of the results.
- In feature engineering, there are few specialized classification models. Most researchers have generally relied on well-known classifiers.

### 1.3. Motivation and the Proposed Feature Engineering Model

In this work, we aimed to address the above-cited research gaps in the field of EEG signal classification. Firstly, while deep learning models are widely used for automatically classifying EEG signals, due to their high classification performance, they often suffer from high time complexities. To tackle this issue, we proposed a feature engineering model designed to perform accurate classification with linear time complexity. This model consists of three phases: (i) feature extraction, (ii) feature selection, and (iii) classification. Our aim is to provide an efficient alternative to deep learning models, which have exponential time complexity due to the training of millions of parameters.

Secondly, there is a significant gap in the availability of explainable models in EEG signal analyses. Most existing models focus solely on classification performance, neglecting the interpretability of the results. To address this, we developed a new symbolic language, termed Lobish, to provide explainable results. Lobish introduces a method for encoding and interpreting EEG signals, facilitating the analysis of brain activity in a clear and structured manner. By using four letters, each representing different brain lobes, Lobish enables neuroscientific explanations for the observed activities.

Thirdly, in feature engineering, there is a lack of specialized classification models tailored for EEG signals. Given that EEG signals consist of multiple channels, we proposed two channel-based models specifically designed to improve classification performance. Additionally, we introduced a specific ensemble model that was applied to the k-nearest neighbor (kNN) algorithm, a well-known and simple distance-based classifier, to achieve high classification accuracy.

We collected a new EEG language dataset from participants whose mother languages are Turkish and Arabic to develop the proposed model.

### 1.4. Novelties and Contributions

Innovations:

- We have proposed a new channel-based transformation model that encodes the signals using the channel indices.
- A new channel-based feature extraction function, termed ChannelPat, has been proposed in this work.
- An EEG language dataset was collected for this work.
- The tkNN classifier has been proposed to achieve higher classification performance.
- A channel-based feature engineering model has been presented to demonstrate the classification ability of the proposed channel-based methods.
- Lobish is a new-generation explainable result generator and a symbolic language.

Contributions:

- A novel EEG language dataset was collected. We used two data collection strategies: (1) listening and (2) demonstration.
- We have proposed a new feature engineering model. Two EEG-specific models have been used: channel-based transformation and feature extraction.
- By proposing Lobish, we obtained explainable results related to the cortical area.

## 2. Material and Method

### 2.1. Material

To collect this dataset, we used 20 commonly used sentences and demonstrated and relayed these sentences to the participants. These sentences are as follows: (1) Hello, welcome. (2) Enjoy your meal. (3) Bye bye. (4) See you again. (5) Never mind. (6) What are you looking for? (7) Where are you from? (8) In which department are you a student? (9) What is your job? (10) Calm down. (11) Get out here. (12) You can trust me. (13) You know nothing. (14) It is your choice. (15) You disgraced us. (16) Hurry up. (17) Good luck. (18) It is better than nothing. (19) Let’s go. (20) You are welcome.

The mother languages of our participants are (i) Arabic and (ii) Turkish. This dataset was collected from 75 participants: 34 (30 males and 4 females) were Arabic speakers, and the remaining 41 (35 males and 6 females) were Turkish speakers. After collecting this EEG dataset, we removed segments with artifacts caused due to blinking, nodding, head movements, speaking, and laughing. We then segmented these EEG signals into 15 s segments. The EEG signals were collected using the Emotiv Epoch X brain cap (Emotiv, San Francisco, CA, USA), which has 14 channels: (1) AF3, (2) F7, (3) F3, (4) FC5, (5) T7, (6) P7, (7) O1, (8) O2, (9) P8, (10) T8, (11) FC6, (12) F4, (13) F8, and (14) AF4. The channel-based methods used were coded based on these 14 channels. In total, 3190 EEG segments were obtained: 1676 from Turkish participants and the remaining 1514 from Arabic participants.

### 2.2. Method

This section introduces the proposed new methods designed to achieve classification and explainable results from the EEG language dataset. The methods include (i) channel-based transformation, (ii) ChannelPat, (iii) tkNN, (iv) Lobish, and (v) the overall EEG language feature engineering model.

#### 2.2.1. Channel-Based Transformation

The aim of the channel-based transformation is to convert the multi-channel EEG signals into a format that allows for the analysis of the relationships and patterns between the different EEG channels. This transformation is a crucial preprocessing step that simplifies the raw EEG data and makes it more suitable for subsequent feature extraction and classification processes.

The objectives of the channel-based transformation are as follows:

The raw EEG signals are recorded across multiple channels, with each channel representing electrical activity from a different region of the brain. The raw data can be complex and challenging to work with directly.

The channel-based transformation simplifies these data by encoding it into a sequence of channel indices, making it easier to analyze and process.

During the transformation, the EEG signal values from all channels at each time point are sorted. The channels are then ranked based on their activity levels (i.e., signal strength).

This ranking provides insight into which brain regions (represented by channels) are most active at any given time, which is crucial for understanding brain dynamics.

The transformation converts the multi-channel EEG signal into a uniform sequence of indices representing the relative activity of each channel. This uniform representation makes it easier to apply machine learning techniques, as it standardizes the input data format.

The transformed signal, now coded from 1 to 14 (for a 14-channel EEG system), is ready for further processing. This step lays the foundation for methods like ChannelPat, which will extract meaningful features from these transformed data.

By encoding the data into indices, it becomes possible to focus on the transitions between channels, which can be indicative of brain state changes.

The presented channel-based transformation aims to convert raw EEG signals into a simplified, standardized format that captures the relative activity of the different channels. This transformation is essential for enabling more effective feature extraction and ultimately improving the performance of classification models in analyzing EEG data. This method is simple and its pseudocode is given in Algorithm 1.
**Algorithm 1.** Pseudocode of the proposed channel-based transformation.**Input:** The used EEG signal with 14 channels (signal) with a length of L. **Output:** The transformer signal created (CS) with a length of 14L.01: count=1; // Counter definition 02: **for** i = 1 to L **do** 03:      data=signal(:,i); // Get values of the channels. 04:      $\left[sd,idx\right]=argsort(-data)$ where argsort(.): sorting function by descending, sd: sorted data, idx: indices of the sorted data. 05:          $CS\left(count:count+13\right)=idx$; // Creating transformer signal 06:          count=count+14; 07: **end for i**

Using this transformation (see Algorithm 1), the transformed signal was created and coded from 1 to 14 since our dataset has 14 channels.

#### 2.2.2. Channel Pattern

For this work, a new feature extraction function has been proposed. This function has been utilized with the channel-based transformation. In this feature extraction function, we coded the channel transition to create a map signal. After that, the histogram of the created map signal was extracted and used as the feature vector.

The primary goal of ChannelPat is to capture the transitions between EEG channels, which are indicative of the dynamic interactions between different brain regions. By analyzing the activity shifts from one channel to another, ChannelPat can reveal patterns associated with specific brain states or cognitive processes.

ChannelPat converts the sequential transitions between channels into a “map signal.” This map signal serves as a representation of the EEG data that emphasizes the temporal relationships between channels, rather than just their individual activities.

The steps of this model are given below.

- S0: Load the signal.
- S1: Apply the channel-based transformation to the signal.
- S2: Divide the transformed into the overlapping block with a length of 2.

(1) $$p^{t}=CS\left(t:t+1\right),\;t\in \{1,2,\ldots ,14L-1\}$$

Herein, $p^{t}$ is the overlapping block with a length of two. We have used these blocks to show transition.

- S3: Create the map signal by deploying base 14 to decimal conversion.
  (2) $$map=14\left(p^{t}\left(1\right)-1\right)+p^{t}\left(2\right)-1$$
- S4: Extract the histogram of the generated map signal.
  (3) $$feat=\theta \left(map\right)$$
  where feat is the feature vector with a length of 196 (=14^2^) and θ(.) is the histogram extraction function.

The four steps outlined above define the proposed ChannelPat feature extraction function.

By using transitions between channels as the basis for feature extraction, ChannelPat contributes to the interpretability of the model. The features it generates can be linked back to specific brain region interactions, which can be crucial for understanding the underlying neural mechanisms and for providing explainable results in neuroscience research.

#### 2.2.3. tkNN

An innovative ensemble classifier has been used in this research, termed tkNN since the t algorithm has been implemented using the kNN [35] classifier. The proposed t algorithm uses a classifier. By changing the hyperparameters of the classifier, additional classifier-based outcomes are created. After that, iterative majority voting (IMV) is applied to these classifier-wise outcomes to create the voted outcomes. In the last phase of the t algorithm, the most accurate outcome is selected as the final outcome.

The primary objective of tkNN is to improve classification accuracy over traditional kNN by exploring a broader range of hyperparameters, such as distance metrics, distance weights, and the number of nearest neighbors (k). By systematically varying these parameters, tkNN generates multiple classifier outcomes, allowing for the selection of the most accurate result.

By implementing a systematic approach to hyperparameter tuning, tkNN aims to automate the selection of the best combination of k values, distance metrics, and weights. This approach ensures that the final model is optimized for performance without relying on manual tuning, which can be time-consuming and less effective.

In this work, the t algorithm has been applied to kNN. We have iteratively changed distances, distance weights, and k values to create more outcomes. In the distance category, L1-norm and L2-norm (Manhattan and Euclidean) have been used. For distance weights, equal, inverse, and squared inverse parameters have been used. Finally, we used values from 1 to 10 for the k value of the kNN. In this aspect, outcomes have been created with the value of 60 (=2 distances × 3 distance weights × 10 k values). Moreover, 58 additional voted outcomes have been generated using IMV [36]. In the last phase, the outcome with maximum accuracy is chosen as the final result.

**Figure 1:**
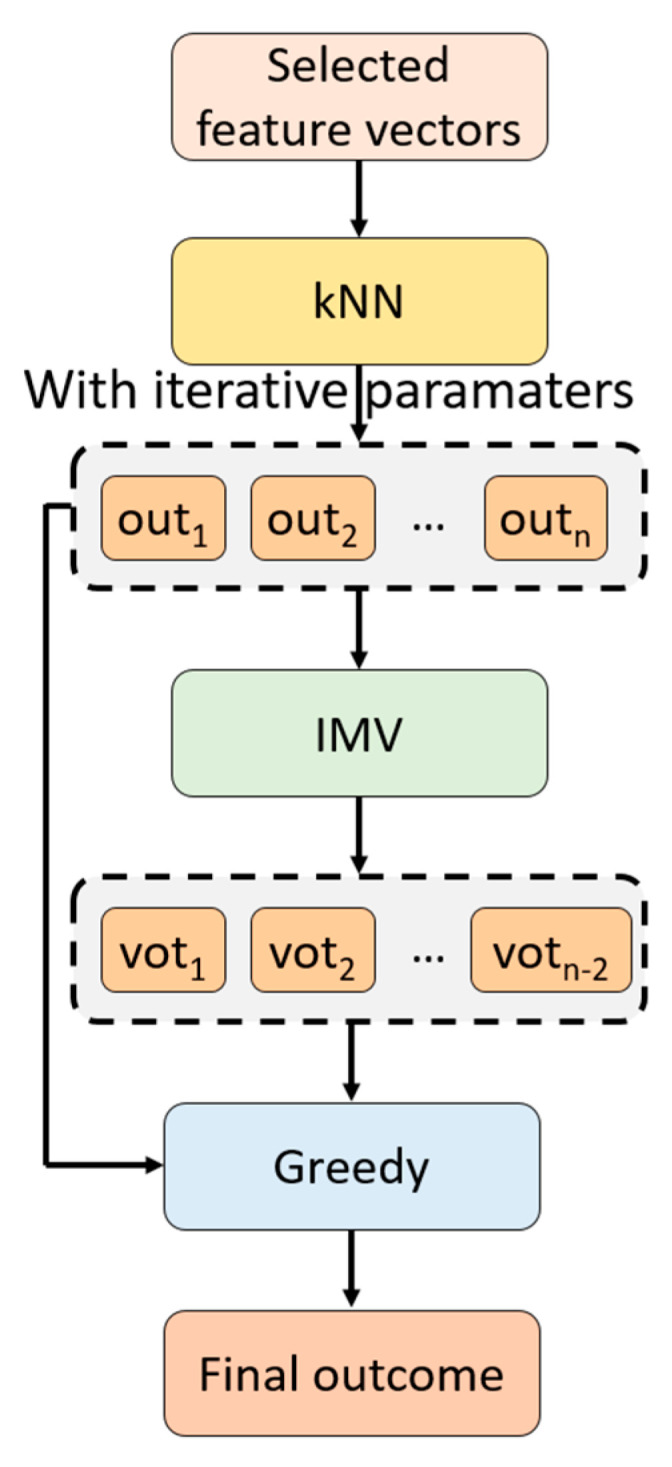
The schematic block diagram of the proposed tkNN classifier. Here, out is the parameter-based outcome and vot is the voted outcome.

To better explain the recommended tkNN classifier, the graphical explanation of this classifier is depicted in Figure 1.

The steps of the tkNN classifier are given below.

- S1: Change the parameters iteratively to create classifier-wise outcomes.
  (4) $$c_{r}=kNN\left(feat,y,k,d,w\right),\;k\in \left\{1,2,\ldots ,10\right\},\;d\in \left\{L1,L2\right\},\;w\in \left\{Equal,Inverse,Squared\;Inverse\right\},\;r\in \left\{1,2,\ldots ,60\right\}\;$$
  where c is the classifier-wise outcome, $kNN\left(.\right)$ is the kNN classifier, and y is the real outcome.
- S2: Apply IMV to the classifier-based outcomes. The mathematical definitions of the IMV algorithm have been given below.
  (5) $$cacc\left(r\right)=\rho (c_{r},y)\;$$
  (5a) ix=γ(cacc)
  (5b) $$v_{h}=\varpi \left(c_{ix\left(1\right)},\;c_{ix\left(2\right)},\;\ldots ,c_{ix\left(q\right)}\right),\;q\in \left\{3,4,\ldots ,60\right\},\;h\in \left\{1,2,\ldots ,58\right\}\;$$

Herein, cacc is the classification accuracy, ρ(.,.) is the classification accuracy calculation function, ix are the sorted indices, ϖ(.) is the mode function, and v is the voted outcome.

In this step, 118 (= 60 classifier-based outcomes + 58 voted outcomes) outcomes were created.

- S3: Choose the final outcome by deploying a greedy algorithm.
  (6) $$cacc\left(60+h\right)=\rho (v_{h},y)\;$$
  (6a) $$\left[maksi,xi\right]=max(cacc)$$
  (6b) $$finout=\left\{\begin{array}{c} c_{xi},xi\leq 60\\ v_{xi-60},xi>60 \end{array}\right.\;$$

Here, maksi is the maximum value, xi is the index of the maximum accuracy, and finout is the final outcome.

The tkNN model explores a wide range of configurations (up to 118 different outcomes), offering a comprehensive analysis of the potential performance of various kNN-based classifiers. This extensive exploration is intended to ensure that the best possible model is selected based on empirical evidence.

The proposed tkNN classifier optimizes classification accuracy, and increases the robustness by using IMV and providing automatic hyperparameter tuning. These make tkNN a powerful classifier.

#### 2.2.4. Lobish

In this study, we proposed an explainable EEG classification method. Therefore, we used the selected features to create a sentence in a new language named Lobish. Lobish is a new-generation method for encoding and interpreting brain activities designed to interpret EEG signals. It uses a symbolic language with four letters corresponding to the four lobes of the brain: F (frontal lobe), O (occipital lobe), P (parietal lobe), and T (temporal lobe). The meanings of these letters are

- F demonstrates cognitive functions.
- P represents sensory processing and spatial awareness.
- T involves auditory processing and memory.
- O indicates visual processing.

Using the defined 4-letter transitions, 16 (=2^4^) transitions have been generated, and the translation of the words with two letters in Lobish is also explained below.

- FF defines sustained cognitive effort.
- TT indicates continuous auditory processing or engaging with memory recall.
- PP depicts ongoing sensory integration and spatial processing.
- OO defines continuous visual processing.
- FT defines the transition from planning or decision-making to recalling information or understanding spoken language.
- FP represents moving from cognitive tasks to integrating sensory information.
- FO depicts the transition from planning or thinking to analyzing visual information.
- TP uses auditory information or memory to assist in sensory processing.
- TO defines recalling visual memories or interpreting visual information based on auditory input.
- PO represents integrating sensory and spatial information with visual processing.
- TF uses auditory or memory information for planning or decision-making.
- PF defines transitioning from sensory information to cognitive tasks.
- OF uses visual information for cognitive processes.
- PT integrates sensory information with memory or auditory processing.
- OT defines associating visual stimuli with memory recall or auditory information.
- OP represents using visual information for sensory and spatial awareness.

Using this language, we obtained results explained by the proposed channel-based model. Moreover, Lobish provides an interpretation of lobe transitions.

#### 2.2.5. Proposed Feature Engineering Model

**Figure 2:**
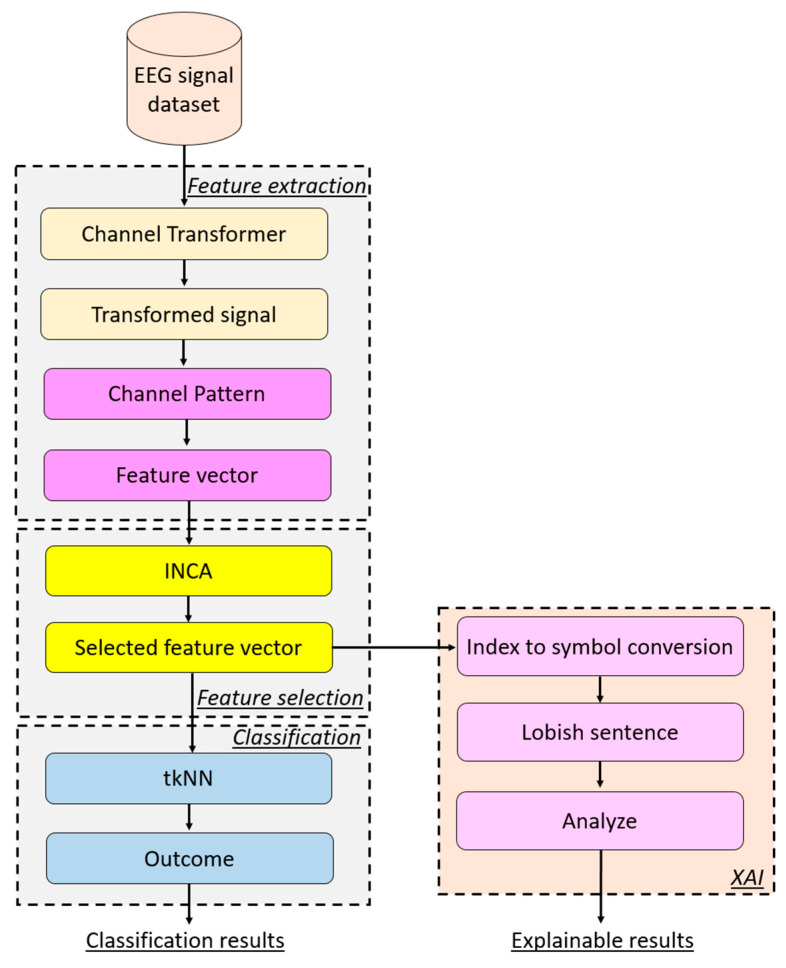
The graphical depiction of the presented explainable feature engineering model.

A new feature engineering model has been proposed to investigate the channel-based transformation and ChannelPat. The proposed feature engineering model has four phases: (1) feature extraction, (2) feature selection, (3) classification, and (4) explainable result generation. The graphical demonstration of the proposed feature engineering model is shown in Figure 2.

The steps are given below to better explain the channel-based feature engineering model.

- Step 1: Apply channel-based transformation to the EEG signal.
  (7) CS=transformer(signal) 

Herein, CS is the transformed signal and transformer is the proposed channel-based transformation. Herein, signal is the input EEG signal with 14 channels.

- Step 2: Extract features by deploying the proposed ChannelPat.
  (8) fv=CP(CS)
  where fv is the feature vector and CP(.) is the proposed ChannelPat. Herein, the length of the feature vector is computed as 196. In this step, 196 (=14 × 14) features have been extracted since the used EEG signal dataset has 14 channels.
- Step 3: Repeat Steps 1–2 until the number of the signals is reached and a feature matrix is created.

**Figure 3:**
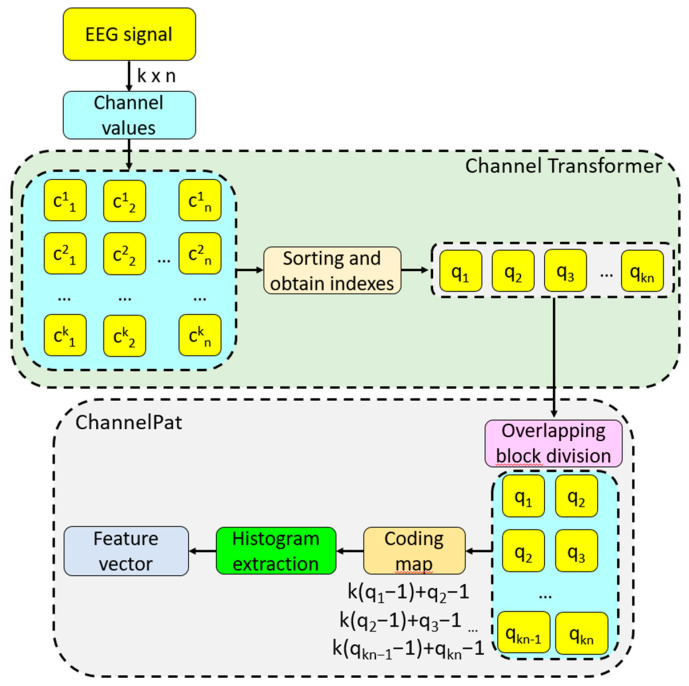
Schematic diagram of channel transformer and ChannelPat (feature extraction of proposed model). c: channel values, k: number of channels, n: length of signal, q: transformed values.

Steps 1–3 have been defined as the proposed feature extraction method of the presented feature engineering model. Moreover, Figure 3 represents the proposed channel-based transformation and ChannelPat.

- Step 4: Apply the INCA [37] feature selector to choose the most informative features.
  (9) sf=INCA(X,y) 
  where sf is the selected feature vector, INCA(.) is the INCA feature selection function, and X is the created feature matrix. By utilizing the selected features, both classification and explainable results have been obtained.
- Step 4 defines the feature selection phase of the proposed feature engineering model.
- Step 5: Classify the selected feature vector by deploying the tkNN classifier.
  (10) res=tkNN(sf,y) 

Herein, res are the results and tkNN(.) is the tkNN classifier.

- Step 6: Extract Lobish symbols by utilizing the indices of the selected features. In this work, we employed a transition table-based feature extraction function, where each selected feature represents a transition between two EEG channels. Consequently, each selected feature corresponds to two Lobish symbols, which are derived based on the specific transitions between the brain lobes represented by those channels. The Lobish symbols provide a symbolic interpretation of the brain’s activity, allowing for both detailed classification and explainable results, as they offer insights into the underlying neural processes associated with the observed EEG patterns. The pseudocode of this step is given in Algorithm 2.

**Algorithm 2.** Pseudocode of the proposed Lobish sentence generation method.**Input:** The indexes of the selected features (id), look up table for Lobish according to used brain cap. LUT = [F,F,F,F,T,P,O,O,P,T,F,F,F,F] **Output:** Lobish sentence (sen)01: c=1; // Counter definition 02: **for** i = 1 to leng(id) **do** 03:      $val_{1}=\left\lceil \frac{id}{14}\right\rceil$; // Compute values for extracting the Lobish symbol 04:      $val_{2}=id\;\left(mod\;14\right)+1$ 05:         $sen_{c}=LUT(val_{1})$; // Extraction Lobish symbols from LUT 06:         $sen_{c+1}=LUT(val_{2})$; // Extraction Lobish symbols from LUT 07:         c=c+2; 08: **end for i**

- Step 7: By utilizing the extracted Lobish sentences, obtain explainable results. These sentences, composed of Lobish symbols, provide a structured interpretation of the brain’s activity, translating complex neural processes into a symbolic language. This approach enables a deeper understanding of the EEG data by linking specific brain lobe transitions to cognitive functions, thereby facilitating both the precise classification and meaningful, interpretable explanations of the observed brain dynamics. In this step, histograms of the symbols and transition tables of the symbols have been computed.

## 3. Experimental Results

This section presents the results of the proposed channel-based EEG language detection model. To implement this model, MATLAB (version 2024a) was used. Firstly, the EEG segments were gathered from participants and segmented, and each signal was stored as a .mat file.

After that, the proposed channel-based model was programmed using various functions: channel transformation, the presented ChannelPat, the INCA feature selection function, and the tkNN classifier. We coded these functions using .m files. The proposed model was implemented using the CPU mode since this model is a feature engineering model with linear time complexity. The parameters of the recommended model are tabulated in Table 2.

**Figure 4:**
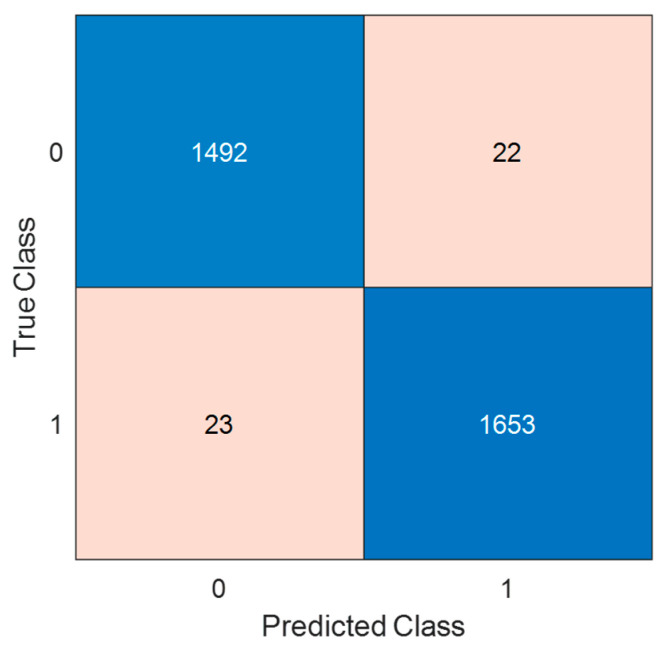
Confusion matrix obtained for proposed channel-based model. 0: Arabic, 1: Turkish.

Using the parameters listed in Table 2, we have implemented the proposed channel-based feature engineering model. This model was designed to solve a binary classification problem, as there are two classes: (i) Arabic and (ii) Turkish. Therefore, classification accuracy, sensitivity, specificity, precision, F1-score, and geometric mean performance evaluation parameters have been used. The confusion matrix of the results was used to compute these performance evaluation parameters, and the computed confusion matrix of the proposed model is shown in Figure 4. Moreover, the performance evaluation metrics are tabulated in Table 3.

As can be seen in Table 3, all performance metrics have attained over 98%.

The second performance evaluation parameter is the time complexity [38]. The big O notation has been used to compute the time complexity of the proposed model.

Channel-based transformation: The time complexity of this transformation is O(N), where N is the length of the signal.

ChannelPat: ChannelPat only uses the conversion from base 14 to a decimal number and extracts a histogram. In this case, the time complexity of this feature extraction function is O(N).

INCA: INCA is an iterative feature selector. Thus, its time complexity is O(S + RC), where S is the time complexity of the NCA feature selector, R is the range of the iteration, and C is the time complexity of the kNN classifier.

tkNN: tkNN is an iterative ensemble classifier. The time complexity is O(PC + V + P), where P is the tested number of parameters or generated predictions, and V is the number of voted outcomes.

Therefore, the time complexity of the proposed channel-based model is equal to O(N + S + RC + PC + V). This result demonstrates that the proposed channel-based EEG signal classification model has linear time complexity.

In this work, we have presented a new channel-based feature engineering model. Specifically, we introduced a transformer for EEG signals that uses the differences in EEG channels to create new-generation features. In this transformer, the INCA feature selector has been used to select the most informative 144 features out of 196 features. Using these selected features, we created a Lobish sentence with a length of 288 (=144 × 2), as each feature of our model has been coded with two channels. The resulting string in Lobish is

“TFTTPOOPPTFFFFPOFTFFOTTFFFFTFTOOFFFFOOPOFFOTPTFOOFFFFFTFFFFFFFTFPPFFPFTOTPFFFFTPTFTPPFTFPOFTFFOFOFFFTOFFFFOFTTOPPFFPFFFFFPOPTFFPFFOFFOTOPFFTPFFOFPPFFFPFFPOFFPFOOPTFFTFFTPOTFPFPTFTOFFPTPTFFFFPFFFPFOTFFFTFFFFTFFOFFPFFFFFOFTFOFFTFFFTFFFFFOFFFTFPFPFFFFPFOFFPPFFOFOFTPFFFPFFFFFFOTFFPFPFOFTFOFO”.

The translation of the above sentence is given below:

- TFTT: Temporal lobe activity indicating memory and auditory processing, and brief cognitive processing, then back to the temporal lobe.
- POO: Transition from parietal to occipital lobes, indicating sensory processing moving into visual processing.
- PPT: Parietal to temporal transition indicating the integration of sensory information with memory.
- FFFF: Sustained frontal lobe activity indicating prolonged cognitive effort and planning.
- POF: Parietal to occipital to frontal transition, indicating sensory and visual information being integrated into cognitive processes.
- TFF: Temporal to frontal transition indicating memory recall being used for planning or decision-making.
- OTT: Occipital to temporal transition, indicating visual information processing leading to memory recall or auditory processing.
- FFFF: Repeated frontal lobe activity, reinforcing cognitive effort.
- TFTOO: Temporal to frontal transition with sustained occipital activity, showing memory integration with visual processing.
- FFFF: Continued cognitive effort in the frontal lobe.
- OOPOFF: Occipital to parietal to frontal transition, indicating visual information moving through sensory integration to cognitive processing.
- OTPTFOO: This complex transition indicates multiple integrations between the occipital, temporal, parietal, and frontal lobes, suggesting intense processing of sensory, memory, and cognitive information.
- FFFFFT: Sustained cognitive effort with a brief switch to the temporal lobe for memory recall.
- FFFFFFT: Continued high-level cognitive processing with brief temporal involvement.
- FP: Simple transition from frontal to parietal lobes, showing cognitive effort translated into sensory integration.
- PFFO: Sensory information is being processed back into cognitive effort and then visual processing.
- FTOTP: Cognitive effort leading to the temporal lobe, then back to occipital and parietal lobes, indicating complex multi-sensory integration.
- FFFFTP: Prolonged cognitive effort with sensory integration.
- TFTPP: Memory recall and cognitive effort leading to sustained sensory processing.
- FTFPO: Cognitive planning integrating memory, sensory, and visual information.
- FFFFO: Sustained cognitive effort with visual processing.
- FOFFF: Visual to cognitive transition showing the integration of visual information into planning.
- TOFFFF: Temporal involvement leading into sustained cognitive effort.
- OF: Simple visual to cognitive processing.
- TPPT: Sensory to memory transitions indicating the integration of external stimuli into memory.
- FFFFP: Prolonged cognitive effort with brief sensory integration.
- FFFOTF: Sustained cognitive effort with visual integration and brief memory recall.
- FFFTF: Continuous cognitive effort with brief memory recall.
- FP: Cognitive to sensory integration.
- FFFFFO: Sustained cognitive effort with visual processing.
- FTFO: Cognitive planning integrating memory and visual processing.
- FFTFF: Cognitive effort with brief sensory and memory processing.
- TPFF: Sensory to memory to cognitive processing.
- FPOFFP: Sensory and visual integration into cognitive processes.
- TFFTFFF: Memory recall leading to sustained cognitive effort.
- FOFFTFF: Visual to cognitive transitions indicating the complex integration of visual information into planning.
- FTFPPF: Cognitive to sensory transitions showing continuous cognitive effort and the integration of sensory information.
- FTOTPFF: Cognitive to memory, visual, and sensory integration showing complex processing.
- FFFFTP: Prolonged cognitive effort with sensory integration.
- TFTPPF: Memory recall leading to sensory processing and cognitive integration.
- TFFPFO: Memory to cognitive, sensory, and visual processing, indicating complex integration.
- FFOTF: Cognitive planning with visual and brief memory recall.
- FFFF: Sustained cognitive effort.
- FOFT: Integration of visual information into cognitive planning and memory recall.
- FOFO: Indicates a task requiring continuous alternation between thinking and a visual analysis, such as reading, planning, or visual problem-solving.

**Figure 5:**
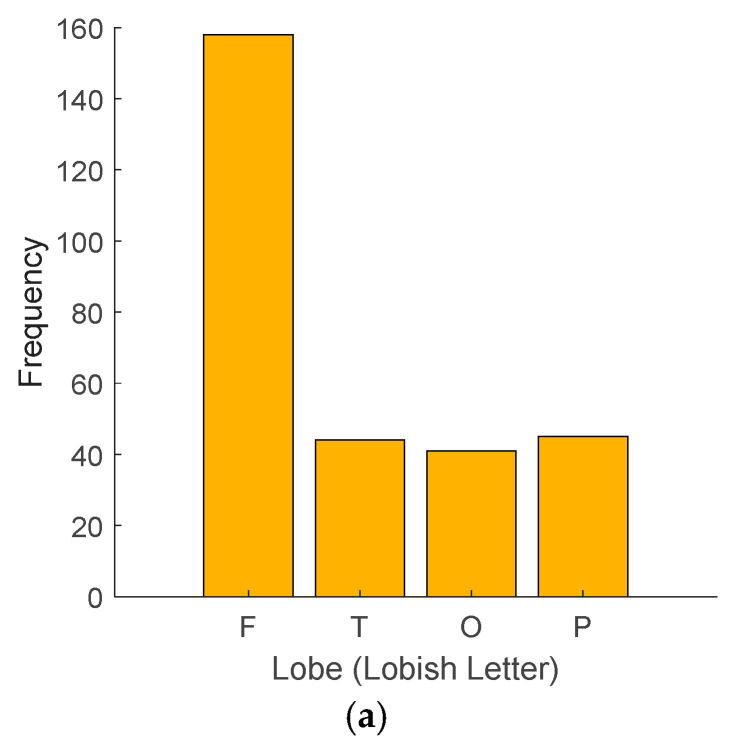

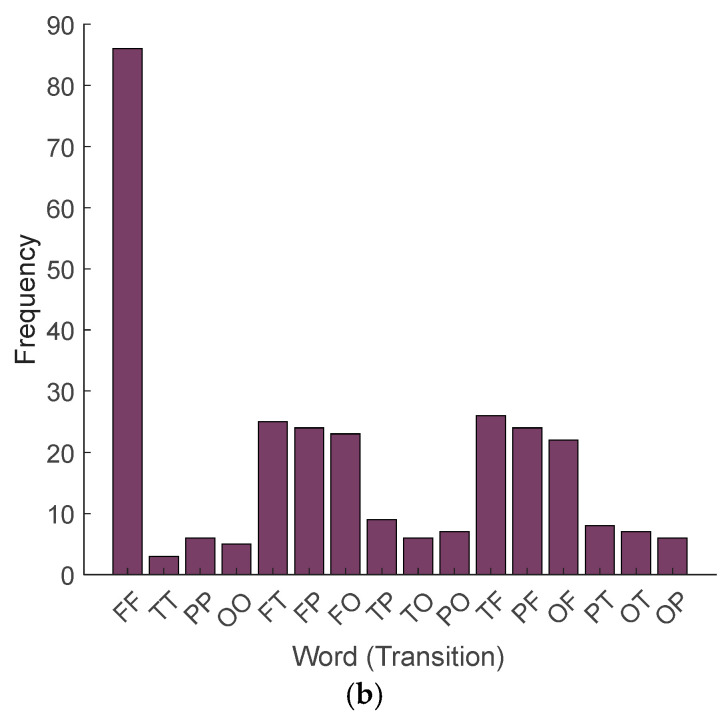
Histogram of (**a**) Lobish sentences and (**b**) transition.

The histograms of the utilized lobes and lobe transitions are shown in Figure 5.

Figure 5a shows 158 frontal, 44 temporal, 41 occipital, and 45 parietal lobe activities. We used demonstration and listening methods to obtain EEG signals during the data collection phase. The obtained Lobish sentence validated this process since the entropy of this sentence, according to Figure 5, is equal to 1.7081. This entropy is relatively high, close to 2 (log₂ 4). This indicates that language detection is a complex process for the cortex. Moreover, the high frontal lobe activity suggests significant cognitive processing. In contrast, the involvement of other lobes highlights the integration of sensory, auditory, and visual information necessary for comprehensive language detection and understanding.

According to Figure 5 (b), the transition frequencies are FF (86), TT (3), PP (6), OO (5), FT (25), FP (24), FO (23), TP (9), TO (6), PO (7), TF (26), PF (24), OF (22), PT (8), OT (7), and OP (6). The dominant transition, FF, with a frequency of 86, indicates a significant and sustained cognitive effort within the frontal lobe. The high frequencies of transitions involving the frontal lobe, such as TF (26), FT (25), FP (24), PF (24), FO (23), and OF (22), suggest that the cognitive processes frequently interact with memory recall, sensory integration, and visual processing. These interactions showcase the complexity of the task, requiring constant switching between thinking, remembering, and perceiving. In contrast, the moderate frequencies of transitions like TP (9), PT (8), PO (7), OT (7), TO (6), and OP (6) indicate a balanced but less frequent integration of sensory and visual information with cognitive functions. The lower frequencies of TT (3) and PP (6) suggest limited continuous auditory and sensory processing within their respective lobes independently. Moreover, the Shannon entropy of the transitions has been computed as 3.3986. This value is close to the maximum entropy of 4 (=log_2_16). Therefore, this entropy demonstrates that the language detection process is a complex process.

**Figure 6:**
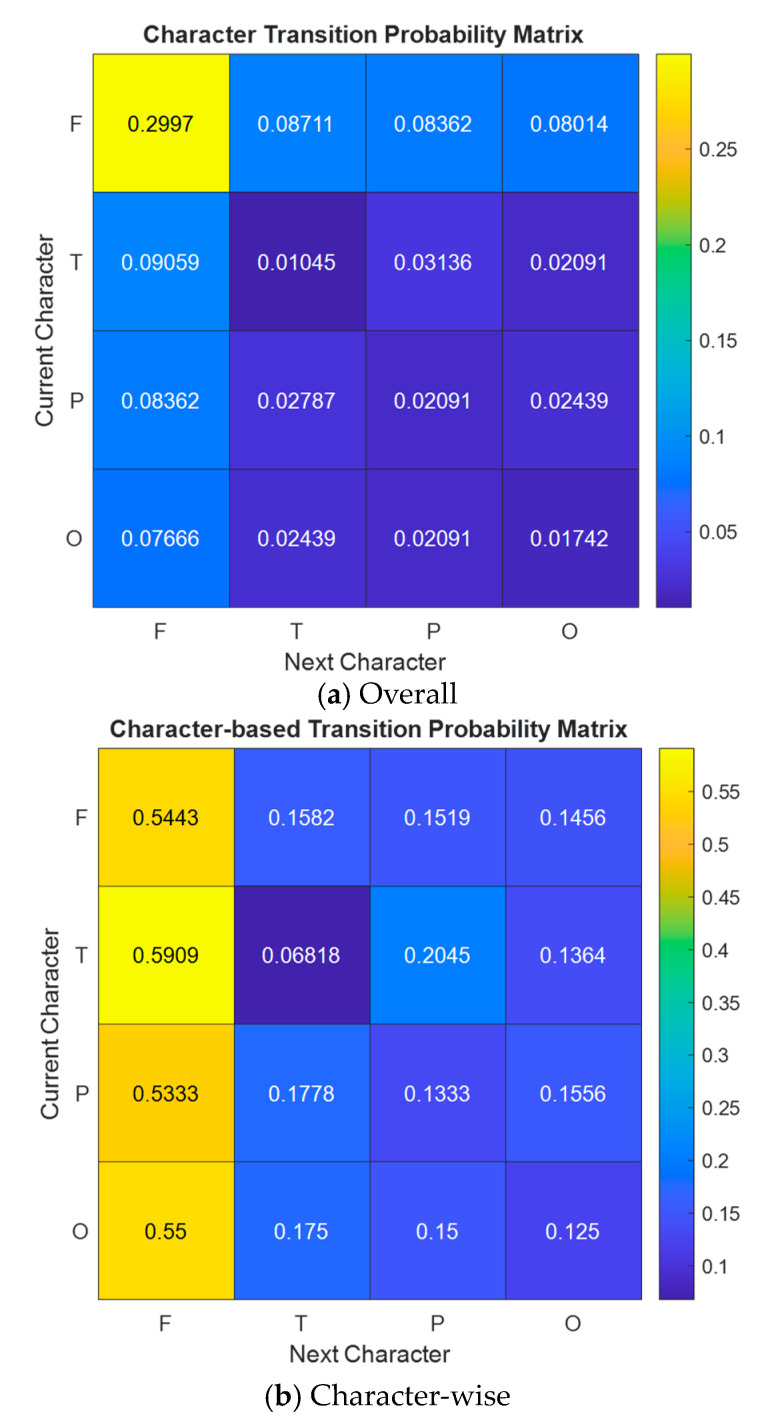
Character transition probability matrix for generated word.

Moreover, the transition matrix of the characters for the generated words has been computed, and this matrix is demonstrated in Figure 6.

Figure 6a indicates that the F state is dominant. The probability of remaining in the F state is higher than in other states, indicating that the F state is relatively stable. The T state is transient: the probability of remaining in the T state is very low, suggesting that the T state quickly transits to other states. The P and O states are also transient: the probabilities of remaining in the P and O states are quite low, indicating that these states change quickly.

Figure 6b denotes that the F state is dominant: the probability of remaining in the F state is higher than other states, indicating that the F state is relatively stable. The T state is transient: the probability of remaining in the T state is very low, suggesting that the T state quickly transitions to other states. The P and O states are also transient: the probabilities of remaining in the P and O states are low, with a higher likelihood of transitioning to the F state.

These analyses help us understand the system’s dynamics using the transition matrix data. Such an analysis can be used to model and understand transition probabilities in EEG signals or other physiological signals.

## 4. Discussions

**Figure 7:**
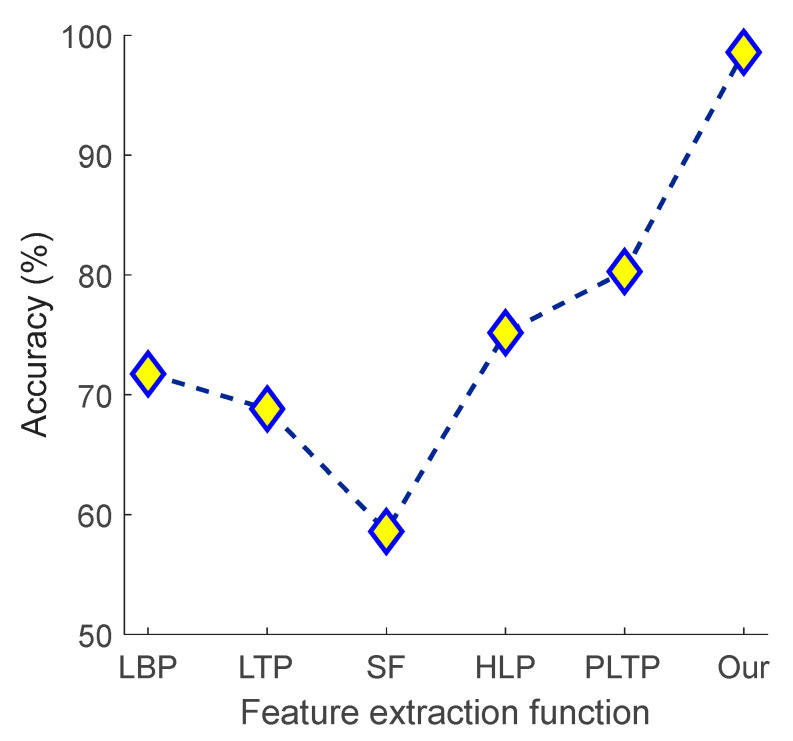
Graph of accuracy (%) obtained versus various feature extraction functions including proposed method.

We compared the proposed model with the commonly used local binary pattern (LBP) [39], local ternary pattern (LTP) [40], statistical feature extractor (SF) [41], hexadecimal local pattern (HLP) [42], and Pascal’s triangle lattice pattern (PTLP) [43]. The results obtained using these methods and the proposed channel-based model are below. We used these feature extraction functions (LBP, LTP, SF, HLP, and PLTP) along with the INCA feature selector and kNN classifier to evaluate the classification performance of various methods on our dataset. Other methods do not include a channel-based transformation method. Therefore, we have used the results from the most accurate channel (the first). The computed classification accuracies for various feature extraction functions are given in Figure 7.

Figure 7 shows that the best other model is the PLTP feature extraction function, which attained 80.28% classification accuracy. In comparison, our model reached 98.59% classification accuracy.

**Figure 8:**
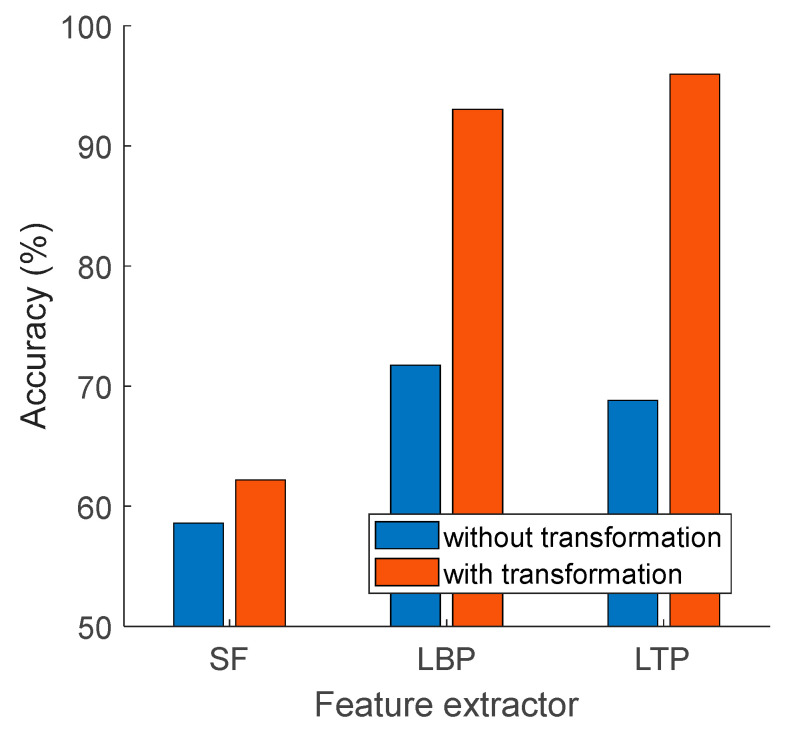
The summary of the effect of the channel-based transformation on the results obtained.

To demonstrate the effect of the proposed channel-based transformation, we applied our EEG transformer to the three least accurate feature extractors: LBP, LTP, and SF. The obtained results are shown in Figure 8.

The proposed channel-based transformation increased the classification accuracies of the SF, LBP, and LTP feature extractors from 58.59%, 71.74%, and 68.81% to 62.19%, 93.07%, and 95.96%, respectively.

**Figure 9:**
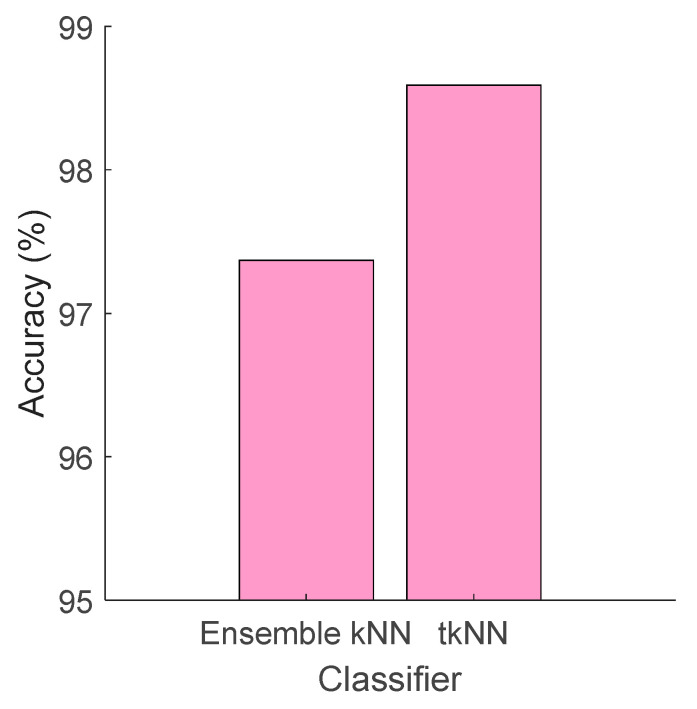
Classification accuracy (%) obtained using ensemble kNN and tkNN classifiers.

The last step of the proposed model is the tkNN method. We have compared the tkNN classifier with ensemble kNN and the results are shown in Figure 9.

The ensemble kNN of the MATLAB classification learner tool attained a 97.37% classification accuracy for our selected feature vector, while the proposed tkNN yielded a 98.59% classification accuracy.

The salient features of this work are given below:

- A novel channel-based transformation function was proposed to obtain higher classification accuracy than the traditional feature extractors like LBP, LTP, and SF.
- The ChannelPat feature extraction function used channel-based transformation to create a map signal, achieving a high classification accuracy of 98.59%.
- The innovative tkNN classifier outperformed the ensemble kNN tool, achieving a classification accuracy of 98.59% compared to 97.37%.
- Lobish, a new symbolic language, was introduced to obtain explainable results.
- The proposed channel-based feature engineering model attained over 98% classification performance.
- This model is a lightweight EEG language detection model since this model has linear time complexity.
- A new EEG language detection dataset was collected, and this dataset includes two languages, which are (1) Arabic and (2) Turkish.
- Lobish has identified the necessity of integrating sensory, auditory, and visual information and high frontal lobe activity for language detection.
- By translating EEG signals into symbolic representations, Lobish has provided deeper insights into the neural processes underlying language perception and processing, paving the way for advanced research in neuroscience and cognitive science.
- The ability to generate Lobish sentences from EEG data opens up new avenues/ways for exploring how different brain regions interact during specific tasks, providing insights that were previously difficult to obtain.
- Lobish serves as a bridge between neuroscience, cognitive science, and artificial intelligence. Its symbolic nature makes it accessible to researchers from different disciplines.
- Lobish can be used to develop personalized learning strategies that align with a student’s cognitive strengths and weaknesses, optimizing learning outcomes.
- The creation of Lobish represents a shift towards a more human-centric approach in EEG analyses.
- Lobish has the potential to transform the way EEG data are used in both research and practical applications, making brain–computer interaction more intuitive and accessible.

## 5. Conclusions

In this study, we introduced a novel channel-based feature engineering model for language detection using EEG signals. Our proposed model, ChannelPat, uses a new channel-based transformation function, which improves the classification performance of traditional feature extractors like LBP, LTP, and SF. In the feature selection phase, the INCA function has been used. To improve the classification performance of our model, the tkNN classifier was proposed and used to obtain classification results. Our developed channel-based feature engineering model attained 98.59% accuracy on the collected EEG language dataset.

We introduced Lobish, a symbolic language with four letters, each representing a different lobe, to increase the interpretability. A sentence was created using Lobish, and by utilizing the obtained sentence, explainable results were generated since Lobish sentences provide relations of the brain lobes for the activities. Lobish highlights the importance of integrating sensory, auditory, and visual information for language detection, with significant cognitive processing evidenced by high frontal lobe activity.

Our results show that the proposed classification model is a transformer of the EEG signal. The proposed model attained high classification performance for language detection.

## 6. Future Directions

- Lobish can be used in neurology, neurosurgery, and neuroscience to understand brain activities better.
- Lobish-based diagnostic and education tools coupled with visualization platforms can be proposed.
- To increase the explainability of Lobish, new letters can be added to represent more specific lobes or parts of the lobes.
- The proposed channel-based transformation can be integrated with deep learning models to achieve higher classification performances.
- The t algorithm can be applied to machine learning algorithms, such as decision trees, naïve Bayes, support vector machines, etc.
- In this work, we proposed ChannelPat to extract features. To obtain more meaningful features, new-generation and effective feature extraction methods can be proposed.

## Figures and Tables

**Table 1:** Summary of state-of-the-art algorithms employed for language/word detection using EEG signals.

Authors	Aim	Dataset	Method	Result(s) (%)	XAI
Sakthi et al. [25]	Native language detection	15 native English and 14 non-native Mandarin Chinese speakers	LSTM-based recurrent neural network	Acc. = 95.0	No
Garcia-Salinas et al. [26]	Automatic Spanish word identification	27 participants and 5 words	Bag of features, codebook and histogram generation, naïve Bayes	Acc. = 68.9	No
Becerra et al. [27]	Automatic Spanish word identification	3 participants and 36 words	Signal decomposition, discrete wavelet transform, linear and non-linear statistical measures, kNN	Acc. ≥ 95.0	No
Vorontsova et al. [28]	Automatic Russian word identification	268 participants and 8 words	Convolutional and recurrent neural network	Acc. = 84.5	No
Bakhshali et al. [29]	Automatic word identification	8 participants and 4 English words	Correntropy spectral density, Riemann distance, kNN	Acc. = 90.25	No
Sarmiento et al. [30]	Automatic vowel identification	50 participants and 5 vowels	Custom-designed CNN (CNNeeg1-1)	Acc. = 85.66	No
Dash et al. [31]	Automatic word identification	15 participants and 6 words	Multivariate fast and adaptive empirical mode decomposition, kNN	Acc. = 60.72	No
Keles et al. [32]	Automatic Turkish sentence identification	20 participants (Turkish and Nigerian volunteers) and 20 sentences (Turkish and English sentences)	Wavelet packet decomposition, TesPat, statistical feature extractor, NCA, kNN, IHMV	Acc. = 95.38	No
Barua et al. [33]	Automatic Turkish sentence identification	20 Turkish participants and 20 Turkish sentences	Multilevel discrete wavelet transform, dynamic-sized binary pattern, statistical feature extractor, NCA, kNN, SVM	Acc. = 95.25	No

Acc: Accuracy, LSTM: Long Short-Term Memory, CNN: Convolutional Neural Network, kNN: k-Nearest Neighbor, SVM: Support Vector Machine, NCA: Neighborhood Component Analysis, IHMV: Iterative Hard Majority Voting.

**Table 2:** Parameters used for the recommended feature engineering.

Method	Parameters
Channel transformer	Sorting function: descending, number of channels: 14, coding: indices.
Channel pattern	Length of overlapping block: 2, base: 14, feature generation function: histogram extraction, length of feature vector: 196.
INCA	Range of iteration: from 10 to 196, selection function: maximum accurate selected feature vector, the number of selected feature vectors: 187, loss function: kNN classifier with 10-fold cross-validation.
tkNN	k value: 1–10, weight: {equal, inverse, squared inverse}, distance: {Manhattan and Euclidean}, number of the generated prediction vectors: 60, validation: 10-fold cross-validation. *IMV:* Sorting function: accuracy by descending order, range: from 3 to 60, number of voted outcomes: 58. Greedy: Selection outcome with maximum accuracy from the generated 118 outcomes.

**Table 3:** The summary of the results obtained for the recommended channel-based feature engineering model.

Performance Evaluation Metric	Class	Result (%)
Classification accuracy	Arabic	-
	Turkish	-
	Overall	98.59
Sensitivity	Arabic	98.55
	Turkish	98.63
	Overall	98.59
Specificity	Arabic	98.63
	Turkish	98.55
	Overall	98.59
Precision	Arabic	98.48
	Turkish	98.69
	Overall	98.59
F1-score	Arabic	98.52
	Turkish	98.66
	Overall	98.59
Geometric mean	Arabic	-
	Turkish	-
	Overall	98.57

## Data Availability

Authors do not have permission to share data.
